# A rare case of primary nodal hemangioendothelioma

**DOI:** 10.3892/ol.2013.1631

**Published:** 2013-10-11

**Authors:** GIUSEPPE DONATO, FRANCESCO CONFORTI, EUGENIA ALLEGRA

**Affiliations:** 1 Department of Pathology, School of Medicine, University Magna Graecia, Campus of Germaneto, Catanzaro I-88100, Italy; 2 Department of Otolaryngology, School of Medicine, University Magna Graecia, Campus of Germaneto, Catanzaro I-88100, Italy

**Keywords:** hemangioendothelioma, lymph node, pathogenesis

## Abstract

Primary vascular tumors of the lymph nodes other than Kaposi’s sarcoma are rare. The present study describes the case of a primary hemangioendothelioma in a cervical lymph node in a patient treated for a carcinoma of the large bowel. Microscopically, the structure of the lymph node was completely substituted by tumoral proliferation, with an architectural pattern consisting of anastomosing vascular channels, a number of which contained papillary projections or a tuft-like structure. This study demonstrates the potential diagnostic errors and hypothesizes the pathogenesis of this type of lesion; a vascular endothelial growth factor (VEGF)/VEGF receptor-3 (VEGFR-3) autocrine loop may be activated.

## Introduction

Primary vascular tumors of the lymph nodes other than Kaposi’s sarcoma are rare (1). Nodal tumors presenting features of tumors of lymphatic endothelial lineage are even more uncommon (2). They occur more commonly in soft tissue and bones, rather than in lymph nodes. Papillary intralymphatic angioendothelioma affect individuals of all ages; however, they are rare in older patients. The present study reports a case of vascular neoplasia with papillation supervening within a cervical lymph node. The tumoral elements exhibited an immunophenotype with characteristics of lymphatic endothelial cells. Other immunohistochemical and clinical findings indicated a new hypothesis for the pathogenesis of this type of rare lesion. Written informed consent was obtained from the patient.

## Case report

A 59-year-old male presented with a painless, slowly growing swelling on the right side of the neck. The clinical examination revealed a 2.0×3.0-cm soft and movable mass, with intact overlying skin of normal color on the upper part of the anterior edge of the sternocleidomastoid muscle.

The patient had a medical history of a moderately-differentiated adenocarcinoma of the sigmoid colon infiltrating the mesorectum in September 2007. The patient received an anterior resection of the rectum and chemotherapy following the folinic acid (200 mg/m^2^), fluorouracil (400 mg/m^2^) and oxaliplatin (85 mg/m^2^) regimen for 12 cycles.

In 2011, the patient presented with lung metastasis, which was treated by left inferior atypical lobectomy and chemotherapy following the folinic acid (200 mg/m^2^), fluorouracil (400 mg/m^2^) and irinotecan (180 mg/m^2^) regimen for 12 cycles.

**Figure 1:**
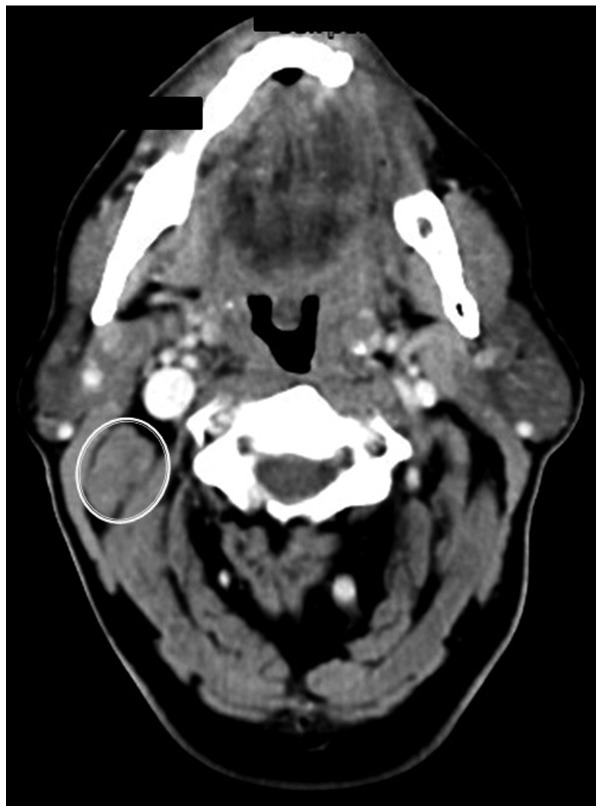
Computed tomography (CT) scan showing a nodular mass in the right cervical space (circle).

In January 2012, during chemotherapeutic treatment, a cervical swelling appeared on a whole-body computed tomography (CT) scan, appearing as a nodular mass of ~3.5×2 cm in the right cervical space between the sternocleidomastoid and scalene muscles (Fig. 1). The remaining areas of the body examined by CT were oncologically negative.

The mass was localized in the neck and was suspected to be a lateral cervical lymph node metastasis. The patient then underwent a neck dissection (levels II–IV) in the Department of Otolaryngology, University Magna Graecia (Catanzaro, Italy).

The patient had no other vascular lesions and continued the previous chemotherapy regimen one month later. The patient subsequently finished the chemotherapy for colon cancer and was oncologically negative at the last follow-up.

The neck lymphadenectomy specimen was accurately sampled. Sections were fixed in 10% neutral-buffered formalin and processed for light microscopy via conventional methods. Routine processing for histological examination included paraffin embedding, sectioning and staining with hematoxylin and eosin.

Sections prepared from the formalin-fixed, paraffin-embedded tissue blocks were used for immunohistochemical analysis. The sections were tested for von Willebrand factor (clone F8/86, 1:50 dilution), CD31 (clone JC70A, 1:40 dilution), CD34 (clone QBEnd10, 1:250 dilution) (all from Dako, Glostrup, Denmark), vascular endothelial growth factor receptor-3 (VEGFR-3; clone KLT9, 1:50 dilution; Leica Microsystems, Wetzlar, Germany), VEGF (clone VG1, 1:50 dilution), podoplanin (clone D2-40, 1:200 dilution), epithelial membrane antigen (clone E29, 1:100 dilution), S-100 protein (polyclonal, 1:2,000 dilution) (all from Dako), CK-AE1/AE3 (clone AE1/AE3, 1:100 dilution; Leica Microsystems) and desmin (clone D33, 1:200 dilution; Dako) using an automated immunostainer (Bond-Max™ Leica Microsystem, Melbourne, Australia).

Sections from tissues samples known to express the detected proteins and sections from tissue samples known not to express the proteins were used as the controls for each immunohistochemical stain.

Of all the regional lymph node chains examined, only the enlarged node shown on the CT-scan revealed the presence of a tumoral infiltration.

**Figure 2:**
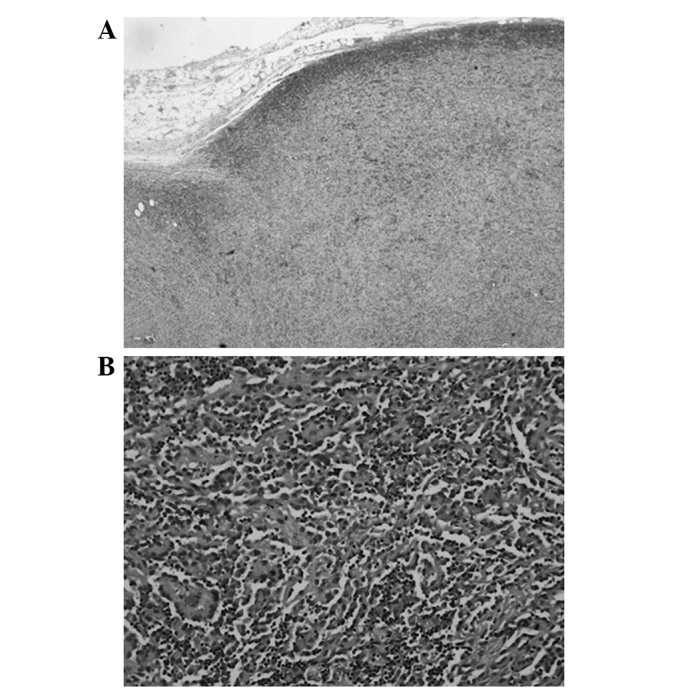
(A) The lymph node structure was lost (HE; magnification, ×10). (B) Lymphatic tissue was completely substituted by tumoral proliferation with an architectural pattern consisting of anastomosing vascular channels, a number of which contained papillary projections (HE; magnification, ×20).

Microscopically, the structure of the lymph node was completely substituted by tumoral proliferation, with an architectural pattern consisting of anastomosing vascular channels, a number of which contained papillary projections or tuft-like structures (Fig. 2).

The papillary sprouts had a hyaline core. The cells exhibiting the papillations were large, with abundant, eosinophilic, cytoplasm; the nuclei were partially crumpled, and their size and shape varied. The chromatin was light and the nucleoli were medium-sized (Fig. 2B).

**Figure 3:**
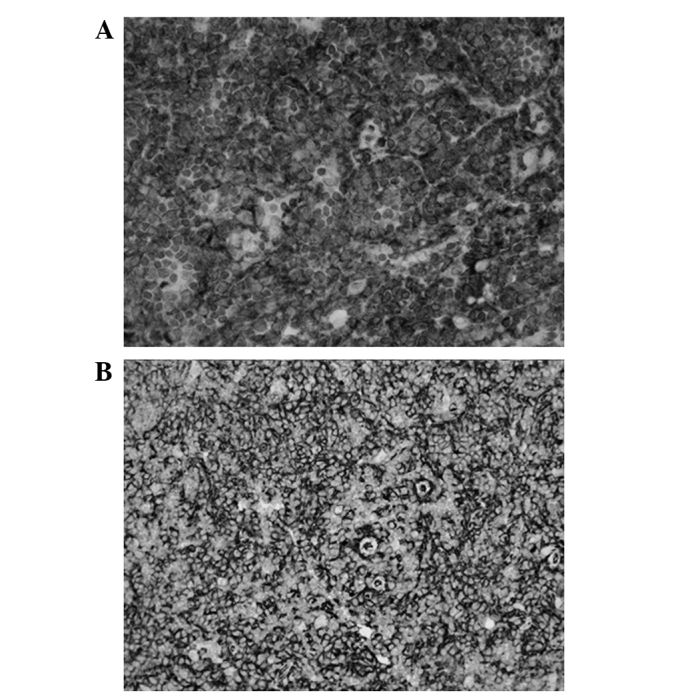
Immunohistochemical positivity for (A) D2-40 (magnification, ×20) and (B) CD31 (magnification, ×20).

**Figure 4:**
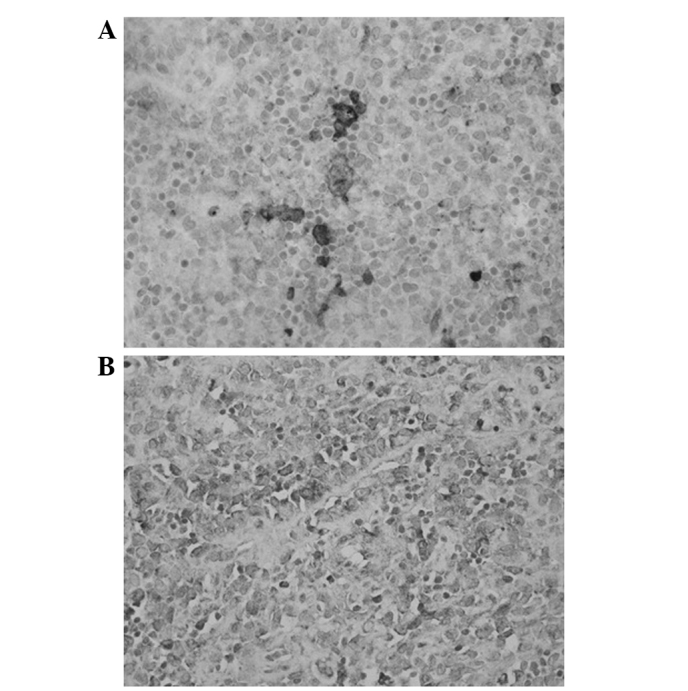
Immunohistochemical focal positivity for (A) vascular endothelial growth factor (VEGF; magnification, ×20) and (B) VEGF receptor-3 (VEGFR-3; magnification, ×20).

The tumor cells were positive immunohistochemically for vimentin, von Willebrand factor, D2-40 (Fig. 3A), CD31 (Fig. 3B), VEGFR3 (Fig. 4A) and focally for CD34 and VEGF (Fig. 4B). The tumoral elements were negative for keratins, epithelial membrane antigen, S-100 protein and desmin. The final diagnosis was that of a hemangioendothelioma.

## Discussion

There are various types of lesions of the head and neck regions that may occur in regional lymph node chains (3). Clinically, non-neoplastic lymphadenopathy has to be differentiated from malignant lymphomas, including Hodgkin’s, peripheral T-cell and non-Hodgkin’s B-cell lymphomas. Patients affected by malignant neoplasms other than lymphomas are often suspected as having metastases when a cervical lymph node is enlarged. The patient examined in the present study was suffering from a carcinoma of the colon with lung metastasis, and a lymph node biopsy was considered necessary for further evaluation of the stage of the disease and the effect of chemotherapy. This patient is the second case in the literature of a primary vascular lesion with Dabska-like features occurring in a lymph node and is also the second case in a patient with a carcinoma of the colon (2). Such an association may be casual, however, other cases have to be described in order to exclude the presence of a link.

Endovascular papillary angioendotheliomas, or Dabska tumors, were first described in 1969 by Dabska (4). These tumors primarily affect the skin of children (5), however, in recent years it has been demonstrated that such a lesion may be present in a wider age range and in more organs than originally described (6). The literature describes a category of ‘similar lesions’ (7), which have two distinctive qualities, namely papillary growth and a lymphatic immunophenotype with positivity for D2-40 (8) and VEGFR-3 (7,9). The pathogenesis of such neoplasms remains obscure. The present study documented immunohistochemically a focal production of VEGF from tumoral cells and the presence of VEGFR-3 in the elements of the neoplasia, so a VEGF/VEGFR-3 autocrine loop may have been activated in the present case, as it is able to occur in several types of solid tumors (10). Differential diagnosis of node tumors should also include rare tumors, including hemangioendotheliomas, particularly when the lymph node structure is substituted by anastomosing vascular channels.
